# Acute transient spinal paralysis and cardiac symptoms following an accidental epidural potassium infusion – a case report

**DOI:** 10.1186/s12871-017-0425-0

**Published:** 2017-10-06

**Authors:** Martin Kreutzträger, Marcel A. Kopp, Thomas Liebscher

**Affiliations:** 1 Treatment Centre for Spinal Cord Injuries, Trauma Hospital Berlin, Warener Straße 7, 12683 Berlin, Germany; 2 Spinal Cord Injury Research, Department of Neurology and Experimental Neurology, Charité – Universitätsmedizin Berlin, Charitéplatz 1, 10117 Berlin, Germany

**Keywords:** Epidural catheter, Potassium, Accidental infusion, Acute spinal cord injury, Complications

## Abstract

**Background:**

To describe a case of an accidental epidural potassium infusion leading to an acute transient spinal paralysis and cardiac symptoms and review the literature on that topic.

**Case presentation:**

We report the case of an accidental infusion of 900 mg potassium chloride 7.45% (KCl) into the epidural space, which occurred during epidural analgesia in a 74-year-old patient suffering from immobilization due to lumbar back pain as well as from a paralytic Ileus. The event was resulting in vegetative symptoms, such as tachycardia and hypertension accompanied by a motor complete tetraplegia (AIS B) sub C2 with respiratory depression. The endotracheal intubation was necessary.

The patient was treated with 40 mg dexamethasone intravenously, as well an epidural lavage with sodium chloride solution 0.9% (NaCl) through the epidural catheter. The neurologic symptoms completely resolved within five days. An elevation of troponin-T values and a reduced left ventricular ejection fraction (LVEF) of 40% accompanied by transient pectanginous pain were documented. An exertional dyspnea remained.

**Conclusions:**

A symptom complex with elevated sympathetic nervous system activity up to a stress cardiomyopathy is possible following epidural potassium infusion. Additionally, generalized pain and muscle spasticity evolve and a progressive acute spinal cord injury syndrome can occur within minutes, accompanied by respiratory depression. Treatment consists of early intensive care and the symptomatic therapy of the associated symptoms, leading in most of the reported cases to a good clinical outcome.

## Background

The use of epidural catheters as a technique for regional analgesia has increased in the last decades [1].

The epidural catheter analgesia should be performed with a combination of a local anesthetic agent and an opioid to ensure a sufficient pain reduction [2]. Advantages over oral opioid analgesics are the significant reduction of opioid side effects such as respiratory depression, interference with the immune system and decrease in intestinal motility. The placement of thoracic epidural catheters can also significantly lower the incidence of postoperative pneumonia [1, 3, 4].

Extreme precautions are necessary when treating patients with epidural catheters. Furthermore, no epidural procedure should be performed if safety mechanisms, such as unique labeling and special connectors of the catheters and infusions, cannot be assured [5].

We report a case of an accidental epidural potassium chloride (KCl) administration due to confusion between an epidural- and a central vein catheter. In addition, we searched pubmed using the terms ‘potassium’, ‘spinal cord injury’ and ‘epidural’ in order to provide a systematic review of the reported clinical symptoms, therapies and outcomes after accidental epidural potassium infusion.

## Case presentation

**Fig. 1:**
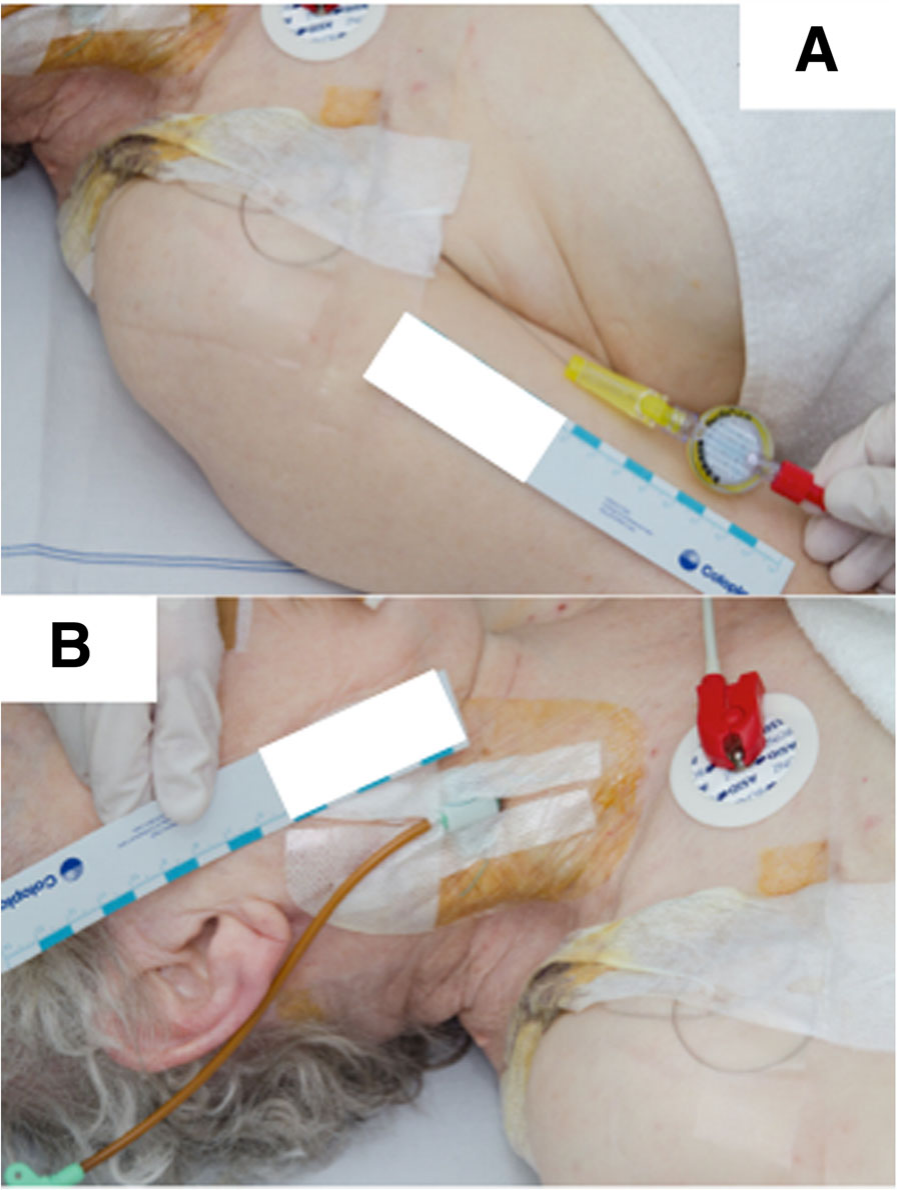
location of the central vein and epidural catheter the epidural catheter that is placed over the right shoulder of the patient is shown in picture **a**. The central vein catheter placed in the right internal jugular vein is shown in picture **b**

A 74-year-old polymorbid patient (Charlson comorbidity index = 5) was admitted to a treatment center for general surgery of an external clinic presenting with lower back pain and and a paralytic ileus [6]. For conservative treatment of acute lower back pain as well as abdominal pain analgesia was needed.. Because of the paralytic ileus the decision against opioid analgesics was made. Therefore the epidural analgesia was performed using a catheter placed at the T 7–8 thoracic level and the infusion of 4 0.2% Ropivacaine at 4 ml/h was performed. It was possible at this point for the patient to be mobilized without pain. Simultaneously, a significant hypokalemia was diagnosed in a routine laboratory examination, leading to the placement of a central vein catheter for intravenous potassium substitution. The catheter was placed into the right internal jugular vein. The end of the epidural catheter was fixed over the right shoulder (Fig. 1a) next to the central vein catheter (Fig. 1b). The infusion of potassium chloride solution (7.45%) with a flow rate of 10 ml/h was accidently connected to the epidural catheter by the nurse. The patient complained early during the infusion about progressive itching over the whole skin surface as well as epigastric pain (see Table 1). Furthermore a motor block Bromage 2 accompanied with tachycardia and hypertensive blood pressure values were noticed. After approximately one hour the accidental connection of the potassium chloride infusion with the epidural catheter was noticed and stopped. A total of 12 ml potassium chloride solution with an equivalent of 800 mg potassium chloride had been epidurally administered. Immediately a lavage with sodium chloride solution 0.9% (40 ml) was administered via the epidural catheter. Simultaneously 40 mg dexamethasone was given intravenously. Despite those therapeutic measures a progressive and ascending flaccid spinal paralysis syndrome with a motor complete tetraplegia accompanied by respiratory depression occurred. For treatment of generalized pain that started in the lower limbs, progressing to the abdomen and thorax and anxiety, 3 mg morphine were administered intravenously. Because of ensuing consecutive respiratory depression and loss of consciousness an endotracheal tube was placed in the intensive care unit. After this the sedated patient was transported via ambulance accompanied by an emergency doctor in our hospital.

**Table 1:** The different symptoms found after epidural potassium infusion in the literature are shown (+ meaning the presence of the symptom)

	Hypertension	Tachycardia	Paresis	Paresthesia	Agitation	Sweating	Perianal itching	Dizziness	Respiratory depression	Leg pain	Spasticity
Kulka [8]	+	+	+	+			+			+	
Litz 1 [17]	+	+	+	+	+	+	+				
Litz 2 [17]	+	+	+	+	+	+				+	
Parodi [11]	+	+	+							+	
Tessler [14]	+	+	+					+	+		
Van der Steeg [15]				+							
Peduto [12]	+	+				+				+	+
Dias [6]	+	+	+	+	+	+			+	+	
Lin [9]	+	+	+	+	+	+				+	+
Shanker [13]			+	+	+				+	+	+
Our case	+	+	+	+	+		+		+	+	

A continuous application of noradrenaline (0.8 μg/kg/min) was necessary at arrival at our hospital to ensure a sufficient blood pressure. Extended diagnostics comprising a whole body contrast-enhanced CT scan, a transthoracic echocardiogram and a MRI scan (cerebral and total-spine) were performed.

**Fig. 2:**
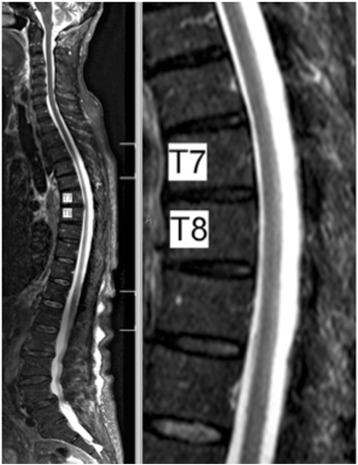
MRI Scan sagittal total-spine MRI scan after the event showing no sign of myelopathy

In the initial MRI scan and the control examination after 24 h no signs of an intraspinal hematoma, a spinal cord lesion, and/or edema could be detected (see Fig. 2).

The CT scan revealed an extended pneumonic infiltration, which was unknown before the event and an inflammatory thickened intestinal wall. Because of a clearly increased leucocyte count of 17.6 gpt/l, the necessity of mechanical ventilation and massive bloody and foamy tracheal secretion a treatment for pneumonia was administered using piperacillin/tazobactam. The antibiotic treatment was converted after 9 days to metronidazol and ciprofloxacin because the ileus required operative treatment. Intraoperatively an obstructive ileus of the small intestine was observed with no need of bowel resection. Antibiotics were continued until discharge.

A transthoracic echocardiogram showed a ejection fraction of 40%. The Troponin T values increased from the initial 376 pg/ml to 937 pg/ml six hours after the potassium infusion. Because specific electrocardiogram changes were missing in the in initial as well as follow-up examination and troponin-t values were declining in subsequent control assessments (every 6 h for the first 48 h), a non-ST-segment elevation myocardial infarction (NSTEMI) without need for intervention was diagnosed. A combination therapy of acetylsalicylic acid and clopidogrel was administered. The weaning from mechanical ventilation was initiated 33 h after admission and was successfully terminated after 91 h. The length of stay at the intensive care unit was 6 days. The neurologic symptoms completely resolved within 5 days. During the further hospital stay until discharge, the patient complained about tolerable exertional dyspnea. After 21 days the patient was transferred to a clinic for geriatric rehabilitation. The therapy of clopidogrel was administered for 12 weeks and a recommendation for a lifelong therapy for acetylsalicylic acid was given.

## Discussion and conclusions

Single cases of accidental epidural infusion of KCl are reported in the literature [7–16]. Reasons described are the confusion between NaCL and KCl as carrier solution and the confusion of infusion syringes [7–16]. Most of the published case reports show a set of symptoms similar to those we observed in our patient (Table 1) [7–15].

Symptoms usually include paresthesia or tingling sensations in the upper and lower extremities, followed by generalized pain. Tachycardia and hypertension have also been reported [7, 9–13, 15, 16].

At latency ranging from a few minutes to a couple of hours muscular paresis occurred [7, 9–12, 14, 15]. In some cases muscle spasticity was documented after a longer latency period [10, 13, 14]. In our case we did not observe any signs of spasticity, which might be due to the early intubation and sedation afterwards.

Parodie et al. reported the additional occurrence of a Takotsubo cardiomyopathy with associated acute cardiac failure and pulmonary edema. They recorded elevated troponin values without ST-elevation, “ballooning “of the left ventricle and no relevant stenosis in the coronary angiography [12]. Likewise, in our patient we also encountered signs comparable with a Takotsubo cardiomyopathy (non-ischemic stress cardiomyopathy) such as reduced LVEF, NSTEMI and pulmonary dysfunction [17]. Because the patient refused to undergo coronary angiography, the accurate differentiation of NSTEMI from an acute myocardial infarction was not possible in our case. Nevertheless, a therapy with acetylsalicylic acid combined with clopidogrel was started.

Dias et al. hypothesized in their case report that the autonomic dysfunction might be caused by the cephalad diffusion of KCl into the cerebrospinal fluid, leading to the depolarization of sympathetic neurons [7]. This could also explain the occurrence of a Takotsubo cardiomyopathy with release of stress hormones like histamines as well as the observed tachycardia and hypertensions.

There is no evidence-based therapy for the physical events triggered by the epidural infusion of potassium (see Table 2). The intravenous application of corticosteroids was often performed as in our case to avoid the possible swelling of the spinal cord (see Table 2) [9, 13, 16, 18]. Liu et al. additionally infused methylprednisolone 100 mg intradurally [18]. It is unclear whether a spinal cord edema occurs at all or whether the spinal cord injury symptoms result from a diffusion of potassium to the intracellular space leading to a signal blockade.

**Table 2:** The different therapy options found after epidural potassium infusion in the literature are shown (+ meaning the usage of the therapy option)

	Symptomatic	Corticosteroids	Epidural lavage	Intubation	Sedation	Remission
Kulka [8]	+	40 mg dexamethasone	NaCl		midazolam	complete
Litz 1 [17]	+					complete
Litz 2 [17]	+				diazepam	complete
Parodi [11]	n/a					complete
Tessler [14]	+	10 mg dexamethasone			diazepam	complete
Van der Steeg [15]		40 mg dexamethasone	100 ml Nacl			complete
Peduto [12]	+	2 g hydrocortisone			midazolam	complete
Dias [6]	+		Replacement of 70 ml spinal fluid with 70 ml NaCl	+		complete
Lin [9]	+		10 ml NaCl		diazepam	complete
Shanker [13]	+			+	diazepam	Paraplegia and death after 6 months
Our case	+	40 mg dexamethasone	40 ml NaCl	+		complete

As shown in Table 2, in some reported cases sodium chloride solution was given via the epidural catheter to dilute the potassium concentration. Kulka et al. suggested that the infusion of a saline solution can lower the potassium concentration in the cerebrospinal fluid [9]. Since already small elevations of the intraspinal potassium concentrations can lead to symptoms, a positive effect of the dilution might be possible. In another case report by Dias et al. 50 ml spinal fluid was removed and replaced with 50 ml NaCl solution. After 5 min the patient showed symptoms of pulmonary edema which required sedation and intubation. Prior to the intubation the neurologic symptoms were tested and a persistent paraplegia with a regression of the sensory level from Th 4 to Th 10 were found. Subsequently, they exchanged again 20 ml spinal fluid with NaCl solution. A complete remission of the symptoms was achieved [7].

With the excerption of the one case by Shanker et al. all other cases in the literature show a complete remission [14]. Based on a low number of cases in the literature, a general therapy recommendation is not possible. Notably, most of the cases presented in the literature reported a complete remission of the neurologic symptoms.

**Fig. 3:**
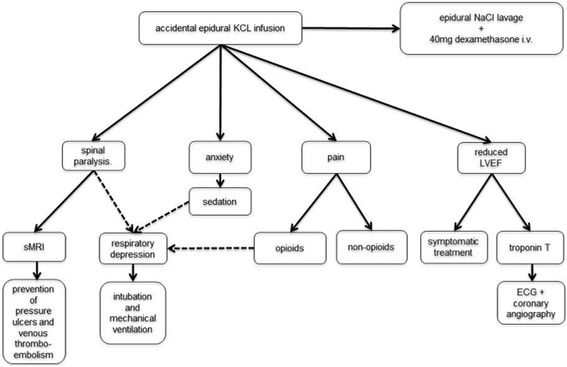
diagnostic and therapeutic work path diagnostic and therapeutic work path

Epidural catheter analgesia should not be performed if a safe handling can not be assured. The accidental infusion of KCl solution into the epidural space represents a serious adverse event with the risk of fatal consequences and necessitates intensive care. A symptom complex with elevated sympathetic nervous system activity (hypertension and tachycardia) up to a stress cardiomyopathy is possible. Additionally, generalized pain and muscle spasticity evolve and a progressive acute paralysis can occur within minutes, accompanied by respiratory depression in need of endotracheal intubation. Further diagnostic measures are indicated to exclude further reasons for this symptom complex. Therefore a total spine MRI scan should be performed to exclude a myelopathy and intraspinal bleeding. Furthermore an echocardiogram is necessary to assess cardiac dysfunction (Fig. 3).

The treatment consists of monitoring and treatment in an intensive care unit and the symptomatic therapy of the associated symptoms, leading in most cases to a good clinical outcome.

## Abbreviations

AIS: American Spinal Injury Association Impairment Scale
CT: Computed tomography
KCL: potassium chloride
LVEF: Left ventricular ejection fraction
MRI: Magnetic resonance imaging
NaCl: Sodium chloride
NSTEMI: Non st elevation myocardial infarction
